# Nanostructured diatom earth SiO_2_ negative electrodes with superior electrochemical performance for lithium ion batteries

**DOI:** 10.1039/d0ra05749e

**Published:** 2020-09-10

**Authors:** Maria Valeria Blanco, Viktor Renman, Fride Vullum-Bruer, Ann Mari Svensson

**Affiliations:** Department of Materials Science and Engineering, Norwegian University of Science and Technology NO-7491 Trondheim Norway maria.v.blanco@ntnu.no; Department of Thermal Energy, SINTEF Energy Research NO-7034 Trondheim Norway

## Abstract

Diatomaceous earth (DE) is a naturally occurring silica source constituted by fossilized remains of diatoms, a type of hard-shelled algae, which exhibits a complex hierarchically nanostructured porous silica network. In this work, we analyze the positive effects of reducing DE SiO_2_ particles to the sub-micrometer level and implementing an optimized carbon coating treatment to obtain DE SiO_2_ anodes with superior electrochemical performance for Li-ion batteries. Pristine DE with an average particle size of 17 μm is able to deliver a specific capacity of 575 mA h g^−1^ after 100 cycles at a constant current of 100 mA g^−1^, and reducing the particle size to 470 nm enhanced the reversible specific capacity to 740 mA h g^−1^. Ball-milled DE particles were later subjected to a carbon coating treatment involving the thermal decomposition of a carbohydrate precursor at the surface of the particles. Coated ball-milled silica particles reached stable specific capacities of 840 mA h g^−1^ after 100 cycles and displayed significantly improved rate capability, with discharge specific capacities increasing from 220 mA h g^−1^ (uncoated ball-milled SiO_2_) to 450 mA h g^−1^ (carbon coated ball-milled SiO_2_) at 2 A g^−1^. In order to trigger SiO_2_ reactivity towards lithium, all samples were subjected to an electrochemical activation procedure prior to electrochemical testing. XRD measurements on the activated electrodes revealed that the initial crystalline silica was completely converted to amorphous phases with short range ordering, therefore evidencing the effective role of the activation procedure.

## Abstract 2

Diatomaceous earth SiO_2_ anodes with superior electrochemical performance are obtained by ball milling, carbon coating and electrochemical activation of SiO_2_ particles.

## Graphical abstract

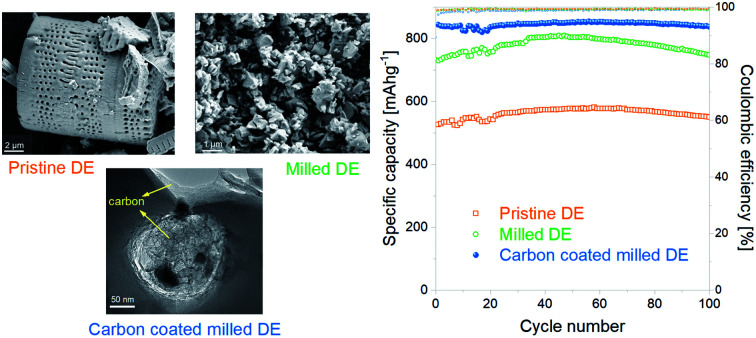

## 1 Introduction

Silica has emerged as a very promising material for negative electrodes in next generation Li-ion batteries (LIBs),^1–9^ displaying a theoretical specific capacity of 1965 mA h g^−1^ and the ability to react with lithium ions in the voltage range between 0.0 and 1.0 V (*vs.* Li^+^/Li). Moreover, it is one of the most abundant materials in the Earth’s crust, which is of key importance from a large-scale application perspective.

The first experimental evidence on the possibility of using silica as the negative electrode in LIBs was reported in 2001, where commercial SiO_2_ nanoparticles were shown to deliver specific capacities of about 400 mA h g^−1^ at 50 mA g^−1^.^10^ Since then, many investigations have successfully enhanced the electrochemical performance of silica anodes by synthesizing nanostructured domains within which silica nanoparticles are combined with conductive components.^1,3,11–13^ In an attempt to increase both the electronic and ionic conductivity of silica, Lener and coworkers synthesized SiO_2_/C composites consisting of a mesoporous silica matrix filled with hard carbon nanorods, achieving a reversible specific capacity of 450 mA h g^−1^ after 300 cycles at a constant current of 100 mA g^−1^.^3^ In different work, Liang and coworkers^11^ implemented a multi-constituent co-assembly approach to prepare SiO_2_/C interpenetrating network structures which exhibited good cyclability, with specific capacities of about 660 mA h g^−1^ after 100 cycles at 200 mA g^−1^, together with excellent rate capability results. In addition to this, hollow SiO_2_ nanocubes with cracked shells have been shown to facilitate lithium movement across the interface while providing extra space to accommodate volume variations produced upon cycling, delivering reversible capacities of 919 mA h g^−1^ after 30 cycles, and coulombic efficiencies close to 95%.^14^ Hence, considering the relatively recent proposal of silica as negative electrode material and the significant improvements already demonstrated, further attempts to close the gap between theoretical and experimental specific capacity values appear as a viable route for developing low-cost, high performance anodes. In this context, the design of nanostructured porous silica particles with conductive coating networks is considered as a very convenient approach.

One of the major natural sources of silica is diatomaceous earth (DE), which consists of fossilized remains of microalgae (diatoms) commonly found in oceans and soil.^15^ Diatom skeletons (frustules) form complex 3D hierarchically nanostructured porous SiO_2_ frameworks that can be transformed *via* covalent/noncovalent functionalization and chemical conversion^16,17^ to different products for a wide variety of catalysis and energy storage applications.^18–22^ Indeed, the unique nanostructure exhibited by the frustules has served as an excellent framework for the production of Si anodes through magnesiothermic reduction of SiO_2_.^23–26^ This method has shown the ability to preserve the DE intricate features, obtaining DE-based nanosilicon anodes with reversible capacities of about 1100 mA h g^−1^ after 50 cycles at 700 mA g^−1^.

In contrast to the vast number of examples where DE is used as a template material, only a few reports have focused on the direct implementation of DE silica into negative electrodes for LIBs.^27–30^ Experimental results have shown that oval-shaped diatoms with an average diameter of 5 μm embedded in a carbonaceous matrix can reach discharge capacities of 460 mA h g^−1^ after 70 cycles at 40 mA g^−1^.^30^ A different work on ball-milled frustules with a mean particle size of 3 μm, reported a capacity of 679 mA h g^−1^ after 50 cycles displayed by carbon coated sea-hauled diatoms. Such capacity is lower than the one exhibited by the uncoated material (723 mA h g^−1^).^29^ The scarce experimental reports on DE electrodes, together with the lack of efficient carbon coating treatments, points out the need for further studies in order to exploit the full potential of this naturally occurring silica anode candidate.

In this work, we present a comprehensive study on the use of DE SiO_2_ as the active material of negative electrodes for LIBs. First, we analyze the benefits of reducing the frustule size to the sub-micrometer level. Then, an optimized carbon coating treatment that significantly enhanced the electrochemical performance of the anode is presented. The superior electrochemical performances achieved with DE SiO_2_ is attributed not only to the particle size reduction and carbon coating but also to an electrochemical activation pretreatment that triggered its reaction towards lithium ions. Due to the crystalline nature of the starting SiO_2_, its reaction with lithium was confirmed by XRD analysis, which evidenced the complete loss of SiO_2_ long-range order crystal structure on cycled electrodes.

## 2 Results and discussion

### 2.1 Uncoated pristine and milled frustules

**Fig. 1:**
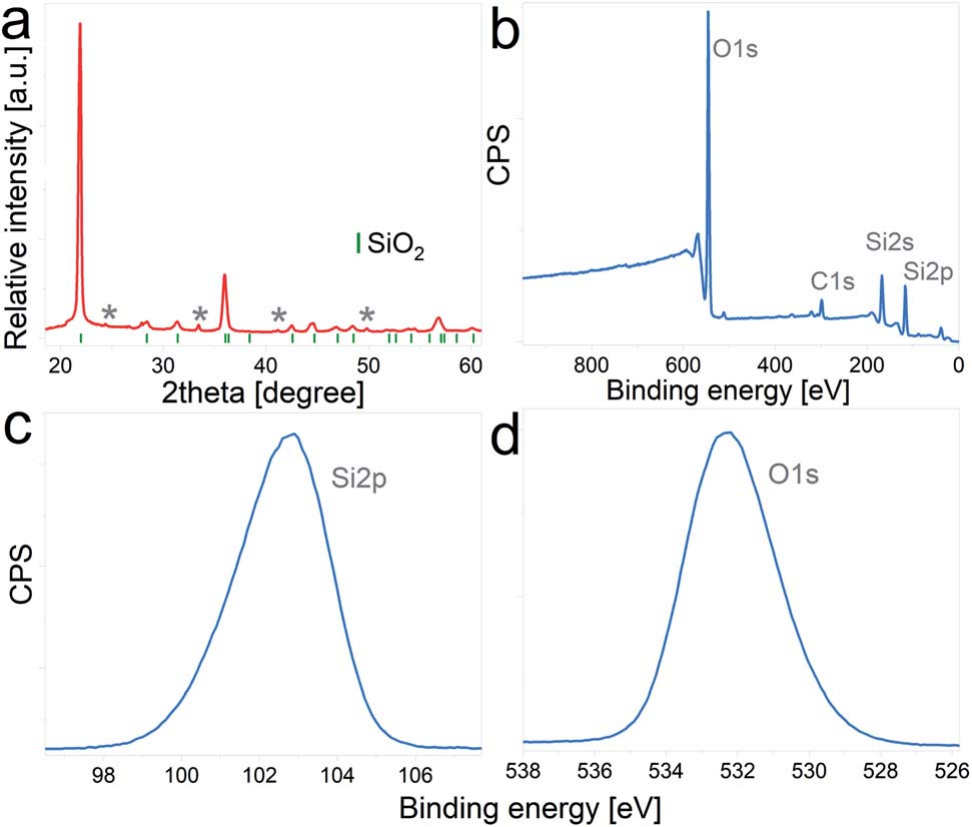
(a) X-ray diffraction pattern of pristine DE particles. Tick marks correspond to reflections of the cristobalite structure, and asterisks indicate Al_2_O_3_ and Fe_2_O_3_ reflections, (b) XPS survey spectrum, (c) Si 2p high resolution spectrum, (d) O 1s high resolution spectrum.

The as-received DE frustules had a faint brown color and a crystalline structure. The XRD pattern, Fig. 1a, shows a tetragonal cristobalite crystal structure (*P*4_1_2_1_2, PDF 00-004-0379) as the main phase, together with low percentages of Al_2_O_3_ and Fe_2_O_3_. The tick marks on the 2θ axis indicate the positions of Bragg reflections belonging to cristobalite, while impurity phases are marked with asterisks. Elemental composition analysis performed by inductively coupled plasma mass spectrometry (ICP-MS) yielded the following atomic percentages (oxygen not included): 86.96% Si, 5.56% Al, 4.04% Fe, 1.33% Ca, 1.19% Na, and less than 1% K and Mg, among others. In addition, the composition and chemical oxidation state at the surface of the frustules (up to 10 nm thickness) were confirmed by XPS. The survey spectrum depicted in Fig. 1b shows the presence of four main peaks at binding energies of 532.3 eV, 284.9 eV, 153.8 eV and 102.9 eV, which are characteristic of O 1s, C 1s, Si 2s and Si 2p, respectively. Quantitative atomic composition analysis, now including oxygen content, displayed the following atomic percentages: 61.03% O, 23.77% Si, 10.42% C, 3.62% Al and less than 1% of Fe, Mg, Ca and Na. The presence of carbon at the surface of the particles is attributed to adventitious carbon contamination. High resolution regional spectra corresponding to Si 2p and O 1s are depicted in Fig. 1c and d, respectively, and show good agreement with previously reported results on SiO_2_ samples.^31–33^

**Fig. 2:**
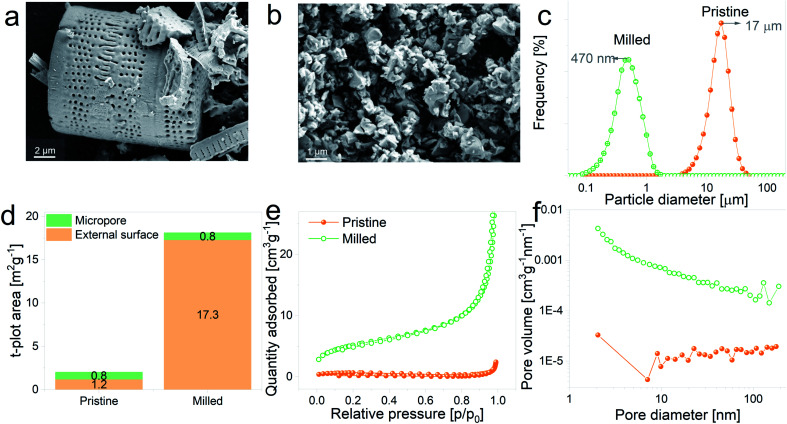
(a and b) SEM micrographs of pristine and ball-milled frustules, respectively, (c) particle size distribution plots, (d) micropore and external surface area plots, (e) N_2_ adsorption–desorption isotherms and (f) pore size distribution curves corresponding to pristine and ball-milled frustules.

With the aim of evaluating the effect of particle size on the electrochemical performance of DE-based anodes, frustules were mechanically milled. SEM images of pristine and ball-milled particles are shown in Fig. 2a and b, respectively. The as received DE exhibits cylindrical shells with periodic arrays of pores, whereas milled frustules consist of irregularly shaped nanoparticles. According to the particle size distribution curves, depicted in Fig. 2c, unmilled frustules display a mean particle size of 17 μm and a narrow size distribution. Ball milling reduced the particle size to 470 nm, leading to an increase in the external surface area, Fig. 2d, from 1.2 m^2^ g^−1^ to 17.3 m^2^ g^−1^. Unmilled and milled particles exhibit type II isotherms, Fig. 2e, which are characteristic of monolayer–multilayer adsorption on non-porous materials. The pore size distribution plot of pristine frustules shows a wide distribution curve, whereas ball-milled frustules exhibit a slightly higher presence of mesopores, as can be observed in Fig. 2f. These results may indicate that the mesopores initially present in the material become more accessible after the ball milling treatment.

Prior to electrochemical testing, electrodes containing unmilled and milled frustules were subjected to an electrochemical activation procedure in order to trigger the SiO_2_ reaction towards lithium. To this end, the electrodes were galvanostatically cycled between 0.002 V (*vs.* Li^+^/Li) and 2 V (*vs.* Li^+^/Li) at a constant current of 50 mA g^−1^. After completion of each half cycle the low and high cut-off voltages were held for 48 h (at 0.002 V) and 24 h (at 2 V), and the entire procedure was repeated 5 times.

**Fig. 3:**
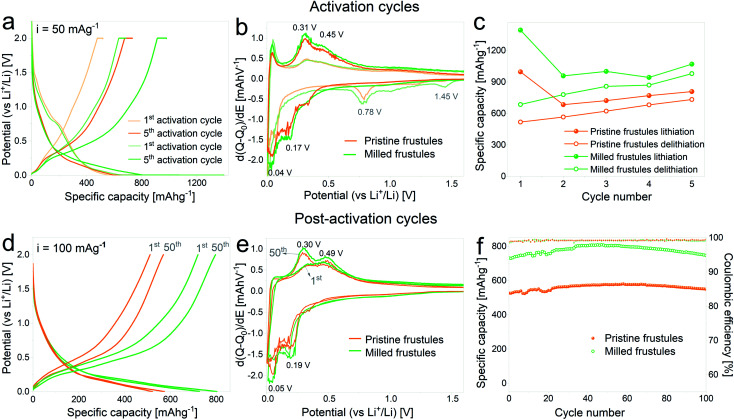
(a and d) Voltage profile curves, (b) and (e) differential capacity plots and (c) and (f) specific capacity against the cycle number of unmilled and milled frustules corresponding to activation (a, b and c) and post-activation (d, e and f) cycling.

In Fig. 3a the voltage profiles corresponding to the 1^st^ and 5^th^ activation cycles on half cells containing pristine and milled frustules are displayed. A gradual increase of the specific capacity upon cycling is clearly observed for both pristine and milled frustules, reaching lithiation specific capacity values of 806 mA h g^−1^ and 1068 mA h g^−1^, respectively. The corresponding differential capacity curves, depicted in Fig. 3b, exhibit two peaks during the first lithiation at 1.45 V and 0.78 V, which have been previously reported and are associated with irreversible reactions and SEI formation.^2,3,14,34,35^ In the 5^th^ cycle the consolidation of two anodic peaks at approximately 0.31 V and 0.45 V, and two cathodic peaks at approximately 0.04 V and 0.17 V, all of them associated with silicon alloying/dealloying reactions,^36,37^ are evidenced. The evolution of discharge capacity (*i.e.* lithiation capacity in a half-cell configuration) and charge capacity (*i.e.* delithiation capacity) against activation cycle number, displayed in Fig. 3c, shows a common trend between pristine and milled frustules in which the specific charge capacities exhibit constantly increasing values. The corresponding coulombic efficiencies range from 50%, for the 1^st^ activation cycle, to 91%, for the 5^th^ activation cycle.

Fig. 3d–f displays the results of galvanostatic cycling at a constant current of 100 mA g^−1^ for activated electrodes. Voltage profile plots corresponding to the 1^st^ and 50^th^ cycles, depicted in Fig. 3d, show the superior electrochemical performance displayed by milled frustules. Interestingly, electrodes made with both unmilled and milled frustules experience a gradual increment in their specific capacities during the first 50 cycles, reaching values of 575 mA h g^−1^ (pristine) and 800 mA h g^−1^ (milled). This behavior was previously reported in SiO_2_ anodes^33^ and can be ascribed to the growth of the electroactive area caused by SiO_2_ electrochemical milling, which was also observed in oxide-based electrodes.^38–40^ The corresponding differential capacity curves, Fig. 3e, show a growing evolution of the anodic and cathodic peaks associated with silicon lithiation/delithiation reactions. Further electrochemical cycling, Fig. 3f, shows that after the 50^th^ cycle, the capacities slightly decrease, reaching values of 550 mA h g^−1^ (pristine) and 750 mA h g^−1^ (milled) after 100 cycles. Coulombic efficiencies on activated electrodes are, in all cases, above 99%. Based on these results, it can be argued that the increase in the exposed surface area and the shortening of Li^+^ diffusion paths, both achieved by decreasing the particle size, have a significant positive effect in enhancing the electrochemical performance of the SiO_2_ electrodes.

It is important to note that the selected upper cut-off voltage of 2 V (*vs.* Li^+^/Li) is high for a real application. Indeed, as can be noticed from the experimental data, there is no significant gain in capacity above *ca.* 1 V. Therefore, the 2 V limit would in practice act as a “hold” step and would provide additional time for Li-ions to be extracted from the material.

### 2.2 Carbon coated milled frustules

Given that silica is an insulator, the electrochemical behavior of the anodes can be improved by increasing the electronic conductivity of the SiO_2_ matrix, which can be achieved by creating a conductive layer covering the particle’s surface. In this case, the coating treatment involved the thermal decomposition of sucrose at the surface of the ball-milled frustules. In addition to reducing the electrode resistance, the carbon layer acts as an elastic shell to buffer volume variations of the active material upon electrochemical cycling.^41^

**Fig. 4:**
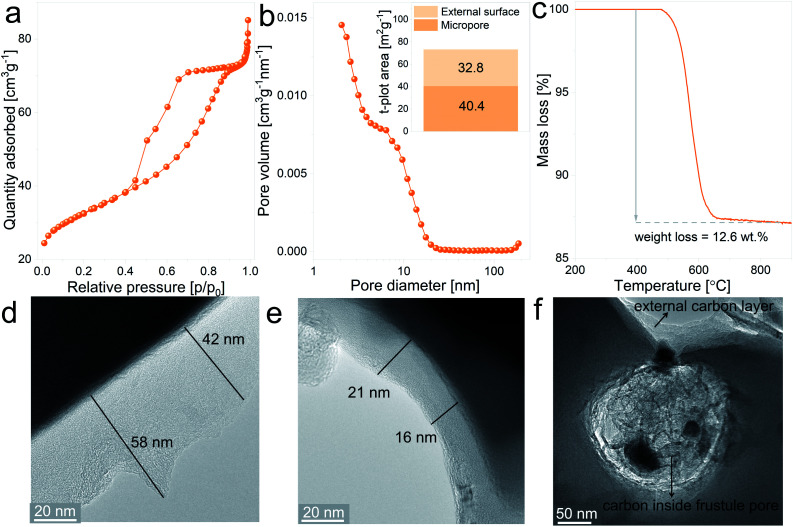
SiO_2_/C composite characterization: (a) N_2_ adsorption–desorption isotherms, (b) pore size distribution curve and insert graph displaying the micropore and external surface area values, (c) evolution of mass loss against temperature under dynamic synthetic air flow, (d, e and f) TEM bright field micrographs.

The SiO_2_/C composites display type IV isotherms, presented in Fig. 4a, with the H2 hysteresis loop, which is indicative of capillary condensation at the mesopores.^42^ The pore size distribution plot, depicted in Fig. 4b, shows a more defined distribution curve in comparison with the uncoated material, with a major fraction of the mesopores exhibiting diameters below 20 nm. Furthermore, the external surface area increases from 17.3 m^2^ g^−1^ for the uncoated material to 40.4 m^2^ g^−1^ for the SiO_2_/C composite, together with a notable increase in the micropore surface area, from 0.80 m^2^ g^−1^ to 32.8 m^2^ g^−1^, respectively. These differences between uncoated and coated SiO_2_ evidences the porous nature of the carbon layer covering the particles. Results from thermogravimetric measurements in which SiO_2_/C composites were heat treated under synthetic air flow are displayed in Fig. 4c and show a mass loss of 12.6 wt%, which corresponds to the actual carbon content on the composite after the coating treatment.

In order to gain insight into the spatial distribution of carbon on the SiO_2_/C composites, samples were examined by TEM imaging. Fig. 4d and e show representative images of the carbon layers covering the milled frustules, displaying variable thicknesses ranging from 16 nm to approximately 60 nm. From the micrographs it can be noticed that thinner carbon layers exhibit a more dense carbon matrix. Interestingly, the pores of silica frustules are filled with carbon, as can be observed in Fig. 4f. The carbon found inside the silica pores exhibit a more porous nature in comparison with the carbon coating layer located at the external surface of the particles. With the chosen carbon coating treatment, the formation of a highly porous carbon layer is expected in the early stages of the annealing process (due to the release of gaseous decomposition products from the carbohydrate), which later evolves to a more compact structure. Based on this, the observed results might indicate that the formation of the coating layer inside the silica pores experiences a “delay” in comparison with the coating layer located at the outer surface of the particles, which are considerably more exposed to the Ar flow, and from which the gas release is expected to be more efficient.

**Fig. 5:**
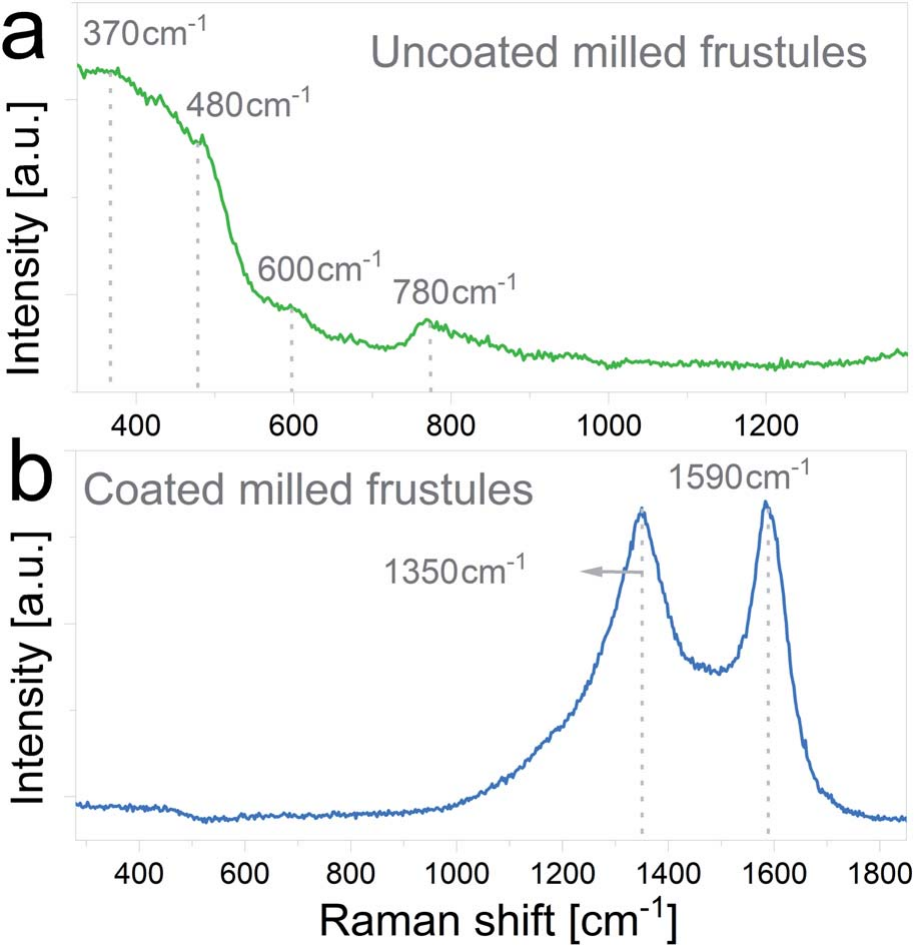
Raman spectra of (a) uncoated and (b) carbon coated ball-milled frustules.

The nature of the carbon coating layer was further characterized by Raman spectroscopy. Raman spectra of uncoated and carbon coated milled frustules are depicted in Fig. 5a and b, respectively. The uncoated material exhibits Raman bands typical of cristobalite structure,^43,44^ with a broad peak near 480 cm^−1^ assigned to the first order Raman scattering of the O–Si–O bond, and bands at 600 cm^−1^ and 780 cm^−1^ associated with the stretching vibration of Si–O. In the case of the carbon coated frustules, the spectrum presents two main peaks at 1350 cm^−1^ and 1590 cm^−1^, which correspond to disorder-induced (D band) and graphitized (G band) carbon, respectively, therefore corroborating the presence of a conductive carbon layer on the SiO_2_/C composites.

**Fig. 6:**
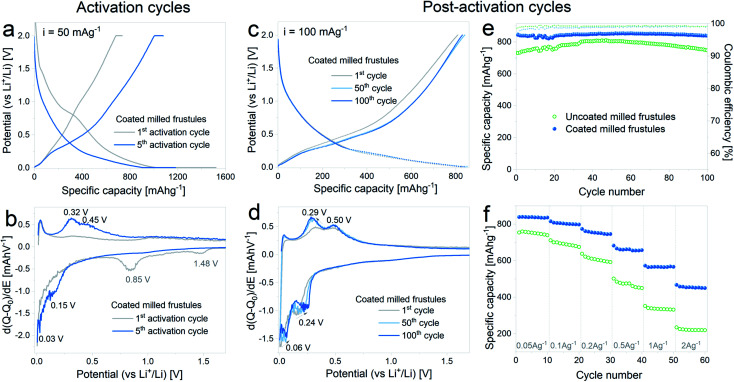
(a) Voltage profile and (b) differential capacity curves of carbon coated milled frustules during the electrochemical activation pretreatment; (c) voltage profile and (d) differential capacity curves, corresponding to the 1^st^, 50^th^ and 100^th^ cycles after electrochemical activation, (e) discharge specific capacity and coulombic efficiency as a function of cycle number, (f) discharge specific capacity displayed at variable current rates.

In Fig. 6 the results corresponding to the electrochemical characterization of carbon coated milled DE electrodes are summarized. Fig. 6a and b show the voltage profile and differential capacity curves, respectively, acquired during the 1^st^ and 5^th^ lithiation–delithiation activation cycles. The first discharge exhibits a specific capacity of 1520 mA h g^−1^ with a subsequent charge specific capacity of 736 mA h g^−1^, entailing an irreversible capacity loss of 52%, which is in line with the results obtained with the uncoated pristine and milled frustules. In the 5^th^ activation cycle, the observed discharge capacity is 1180 mA h g^−1^, while the specific charge capacity is 1080 mA h g^−1^. The corresponding differential capacity curves show the development of the anodic and cathodic peaks associated with silicon lithiation/delithiation, as was previously observed with the non-coated electrodes.

In Fig. 6c and d the curves corresponding to the 1^st^, 50^th^ and 100^th^ cycles acquired upon galvanostatic cycling of the activated electrodes are depicted. The voltage profiles show a change in the slope between the 1^st^ and 50^th^ cycles, and no further changes are evidenced in the subsequent cycles, as observed in the corresponding differential capacity curves. Reversible specific capacity against cycle number plot, depicted in Fig. 6e, shows a stable value of about 840 mA h g^−1^ at a constant current of 100 mA g^−1^, which remains stable up to 100 cycles. In comparison with the uncoated material, the SiO_2_/C composite exhibits not only higher capacity values but also more stable cycling. Results from variable current tests, Fig. 6f, show a significant improvement on the electrode response, with discharge capacities increasing from 220 mA h g^−1^ (uncoated) to 450 mA h g^−1^ (coated) at 2 A g^−1^.

The results indicate that the carbon coating is efficient in providing improved electronic conduction through the porous electrode, without compromising the transport of Li^+^ ions towards the surface. The latter is most likely related to the porous structure of the carbon coating itself, as evidenced in Fig. 4. It is worth mentioning that the reported electrochemical performance, in terms of specific capacity, cycling stability and rate capability is remarkably superior to others reported for similar systems,^27–30^ hence demonstrating the importance of electrochemical activation in combination with adequate carbon coating.

The differential capacity plots presented in this work evidenced the Si alloying/dealloying footprint on activated electrodes, thus indicating that Li-ion reactivity towards SiO_2_ does not involve intercalation, alloying/dealloying or conversion processes in which reversible lithiation/delithiation reactions are the principal source of the reversible capacity.^45,46^ Instead, SiO_2_ would act as a convertible oxide^47–50^ undergoing irreversible conversion reactions,^2,51^ through which lithium silicates, Li_2_O and electroactive Si domains are produced. The *in situ* formed Si would then further react reversibly with Li-ions and would constitute the major contributor to the overall reversible capacity of the electrode.

Although the mechanism through which SiO_2_ is reduced to Si remains unclear, Lepoivre *et al.*^52^ described a method to identify the SiO_2_ lithiation route among certain proposed reactions. As Si lithiation/delithiation is a reversible process, the method is based on determining the increase in the specific capacity associated with a given irreversible capacity loss of a selected cycle. The primary assumptions of the model are as follows: (1) SEI formation and side reactions are negligible, (2) SiO_2_ particles fully react, and (3) the produced Si is fully lithiated. In view of this, the first cycle was excluded from the analysis.

**Fig. 7:**
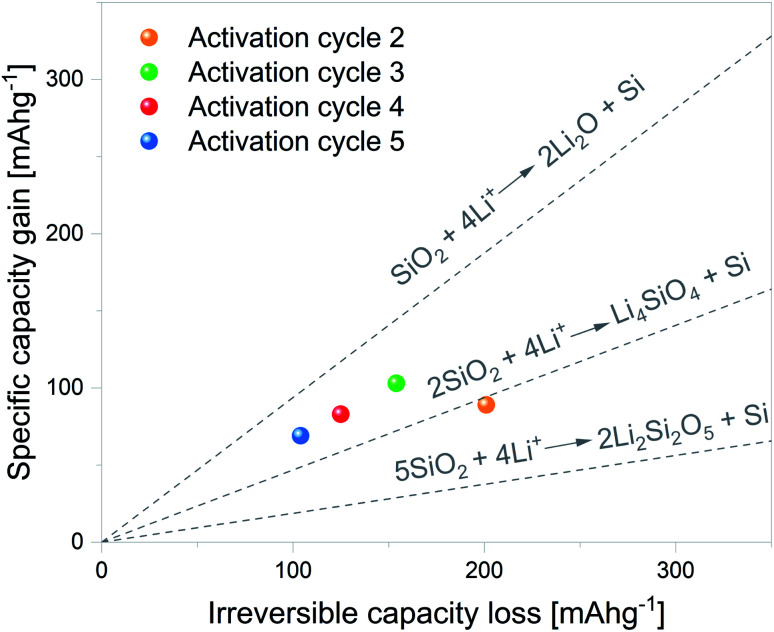
Gain in specific capacity against irreversible capacity loss for activation cycles 2 to 5. Dashed lines indicate the theoretical gains in specific capacity of the considered SiO_2_ conversion reactions.

The considered SiO_2_ conversion reactions are marked on the graphs in Fig. 7, together with their expected increase in specific capacity. Therefore, the experimental results can be directly compared with theoretical values and the SiO_2_ conversion route can be estimated. The data corresponding to electrochemical activation cycles 2 to 5 are plotted within the graph. It can be noted from the plots that the 2^nd^ activation cycle presents a specific capacity gain in line with SiO_2_ conversion to Li_4_SiO_4_ and Si. The following activation cycle presents specific capacity gain values between the dashed lines delimiting SiO_2_ conversion to Li_4_SiO_4_ and Si and SiO_2_ conversion to Li_2_O and Si. These results indicate that both reactions can partially contribute to the overall increase in specific capacity.

### 2.3 Post-activation analysis

In an attempt to demonstrate the irreversible conversion the crystalline SiO_2_ to other phases, the progression of SiO_2_ lithiation was analyzed by X-ray diffraction. To enhance the SiO_2_ signal, new electrodes were prepared with a silica : carbon black : binder ratio of 75 : 15 : 10.

**Fig. 8:**
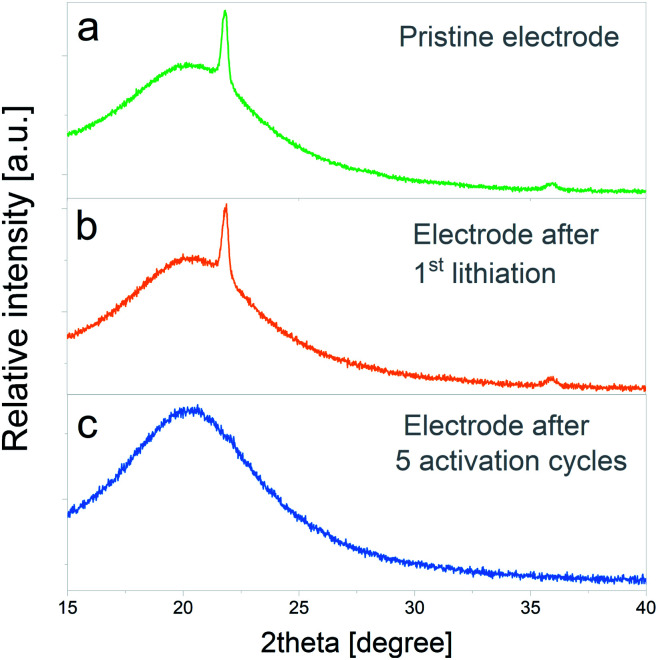
XRD patterns corresponding to: (a) pristine electrode, (b) electrode after 1^st^ lithiation and a 48 h potentiostatic hold step, (c) electrode after 5 electrochemical activation cycles.

In Fig. 8a the XRD pattern of the pristine electrode is depicted, in which the main peak of cristobalite at 2θ = 21.8° and a lower intensity peak at 2θ = 35.9° can be distinguished. The bump on the intensity centered at 2θ = 20.2° corresponds to the signal of the XRD inert atmosphere sample holder. Fig. 8b displays the XRD pattern of an electrode subjected to one lithiation cycle and 48 h potentiostatic hold step. No significant modifications on the initial crystalline SiO_2_ structure are evidenced at this stage. On the other hand, the XRD pattern of an electrode subjected to the entire electrochemical activation pretreatment (5 full cycles) shows the complete disappearance of cristobalite reflections, which indicates that the silica has been converted to amorphous phases. The complete amorphization of electrode materials due to irreversible conversion reactions was previously observed in other systems^40^ and, to the best knowledge of the authors, the presented results would constitute the first evidence of its occurrence during SiO_2_ electrochemical cycling.

## 3 Experimental

### 3.1 Material characterization

The elemental composition of pristine frustules was determined using inductively coupled plasma-mass spectrometry (ICP-MS). Particle size of pristine and milled frustules was measured by laser diffraction (Horiba Partica LA-960 Laser Scattering Particle Size Distribution Analyzer). Carbon content in the composites was determined by thermal gravimetric analysis, using a TGA/DTA probe (NETZSCH STA 449 Jupiter). The material morphology was examined by SEM (FE-SEM Zeiss Ultra 55). Transmission electron microscopy micrographs were collected using a JEM-2100 Transmission Electron Microscope with LaB_6_ filament. N_2_ adsorption–desorption isotherms were measured at 77 K using a Tristar 3000 Surface Area and Porosity Analyzer. Prior to the measurements, samples were out-gassed under vacuum at 250 °C for 12 h. Raman spectra were recorded at room temperature (Alpha300M confocal Raman microscope, WiTec), using an excitation wavelength of 532.1 nm. XRD measurements were performed using a Bruker D8 A25 DaVinci X-ray Diffractometer with Cu Kα radiation.

### 3.2 Preparation of SiO_2_/C composites

Ball-milled diatomaceous earth SiO_2_ was carbon coated with 40 wt% sucrose (Sigma Aldrich) using the following procedure: (1) dissolution of sucrose in distilled water, (2) addition of SiO_2_ particles, (3) 30 min ultrasonication, (4) 2 h of continuous stirring on a hot plate at 60 °C, and (5) manual grinding with an agate mortar. The resulting powder was loaded into an alumina crucible and annealed under Ar flow at 850 °C for 6 h. The carbon coating treatment parameters were selected based on preliminary results in which silica particles were coated with sucrose contents ranging from 20 wt% to 80 wt%, and heat treated at temperatures between 650 °C and 1200 °C.

### 3.3 Electrode preparation and characterization

Slurries containing 50 wt% SiO_2_ (Sigma Aldrich) or SiO_2_/C composites, 35 wt% carbon black (Timcal C-NERGY™ C65) and 15 wt% sodium alginate binder (Sigma Aldrich) were ball-milled at 25 Hz for 45 min (Retsch Shaker 400 MM) and tape casted onto 18 μm thick Cu foils (Schlenk Metallfolien). The resulting electrodes presented a mass loading of 0.5–0.6 mg cm^−2^ and a thickness of 10 μm. Electrodes were dried under vacuum overnight at 120 °C and then transferred to an Ar-filled glove box for further assembly into coin cells, using lithium foil (0.75 mm thick) as the counter electrode and 1 M LiPF_6_ in 50 : 50 vol% EC : DEC electrolyte (Sigma Aldrich).

The electrochemical performance of the electrodes was evaluated by performing galvanostatic charge–discharge tests between 0.002 V and 2 V *versus* Li^+^/Li, at 50 mA g^−1^ and 100 mA g^−1^. Rate capability tests were performed at variable currents of 50 mA g^−1^, 100 mA g^−1^, 200 mA g^−1^, 500 mA g^−1^, 1000 mA g^−1^ and 2000 mA g^−1^ using a Biologic potentiostat/galvanostat. All measurements were carried out at a constant temperature of 20 °C. The capacity values reported in this work include the contribution from carbon black, which was previously determined,^29^ and corresponds to approximately 96 mA h g^−1^ of the overall capacity of the electrodes.

## 4 Conclusions

In this work we investigated the use of crystalline 3D porous structured silica derived from diatomaceous earth as an anode material for Li-ion batteries. Pristine and ball-milled frustules were subjected to an electrochemical activation procedure, which allowed to reach discharge specific capacities close to 550 mA h g^−1^ and 750 mA h g^−1^, respectively, after 100 cycles at a constant current of 100 mA g^−1^. Differential capacity curves evidenced the presence of anodic and cathodic peaks representative of silicon lithiation/delithiation on activated electrodes.

Ball-milled frustules were carbon coated by soaking the silica particles into a solution containing 40 wt% sucrose, followed by an annealing treatment at 850 °C. The resulting SiO_2_/C composites displayed an actual carbon content of 12.6 wt%, in the form of a rather compact nanosized carbon layer covering the external surface of the particles, and a more porous type of carbon filling the original pores of the silica particles. The carbon coating had a remarkable positive effect on the electrochemical performance of the silica anodes. SiO_2_/C composites achieved a stable discharge specific capacity of 840 mA h g^−1^ after 100 cycles at 100 mA g^−1^ and rate tests showed discharge capacities of 450 mA h g^−1^ at 2 A g^−1^ displayed by the SiO_2_/C composites, in comparison with 220 mA h g^−1^ at 2 A g^−1^ achieved by the uncoated frustules.

The XRD characterization on electrochemically activated electrodes revealed that the initial crystalline silica structure completely vanishes after SiO_2_ electrochemical activation, therefore indicating that silica is completely converted to amorphous phases with only short-range ordering.

## Conflicts of interest

There are no conflicts to declare.

## Supplementary Material
